# Estimation of life expectancy of patients diagnosed with the most common cancers in the Valparaiso Region, Chile

**DOI:** 10.3332/ecancer.2017.713

**Published:** 2017-01-17

**Authors:** C Taramasco, K Figueroa, Y Lazo, J Demongeot

**Affiliations:** Escuela de Ingeniería Civil en Informática, Universidad de Valparaíso, 2362905, Chile

**Keywords:** statistical methods, prevalence of cancer, cancerous tumours

## Abstract

**Background:**

The 1000s of people who die from cancer each year have become one of the leading causes of death among the Chilean population, placing it as the second cause of death in the region of Valparaiso between 1997 and 2003. Statistics have provided different measures regarding the life expectancy of cancer patients which have resulted in being useful to establish courses of action for prevention and treatment plans to follow.

**Methods:**

Data was extracted from the cancer module of the Epidemiology Assistance System (SADEPI for its initials in Spanish) which stores information about cancer cases in the provinces of Valparaiso and Petorca. The survival period is defined as the difference in days between the date of occurrence and the date of death of the patient by separating the data into quartiles.

**Results:**

The more frequent cancers in the region of Valparaiso behave similarly to global behaviours of the disease. The majority of affected patients are around 65 years of age which progressively lowers its occurrence in younger adults under the age of 45.

**Conclusions:**

Further efforts are required for early detection and timely access to treatment for cancer patients. Statistics are an important support in achieving this.

## Introduction

The changing lifestyle of the population and exposure to risk factors has led to the emergence and increasing cases of chronic, non-communicable diseases such as cancer.

In most developed countries, cancer is the second cause of death after circulatory diseases [1–3]. Because of population growth and ageing, it is estimated that global cancer mortality will increase by 45% by the year 2030, and the number of new cases will go from 11.3 million in 2007 to 15.5 million in 2030.

In Chile, according to several studies conducted since the end of the last century, malignant tumours are the second leading cause of death. Published data on the occurrence of malignant tumours in the last two decades of the 20th century in Chile, revealed an increase in mortality rates from 98–118 per 100,000 population [5]. These mortality rates have continued to increase up to 133 and 135.3 deaths in 2009 and 2010 respectively [6–7]. The publication “*Cancer Status in Chile 2000–10*” reveals that the Valparaiso Region is the most affected area. In this study 13 out of 16 types of analysed cancers presented rates higher than the national average rates [7]. This fact suggests that continuous studies on cancer prevalence in the Valparaiso Region are still needed to develop appropriate prevention policies.

Statistics have provided various measures regarding the life expectancy of cancer patients [8–9]. For example, according to the National Cancer Institute of the United States (NCI), the index of relative five year survival for women diagnosed with breast cancer during the period 2001–7 was 89% and for patients diagnosed with lung cancer during the same period was 16% [10]. Therefore only 16% of patients diagnosed with lung cancer survived more than five years in relation to people without cancer. This displays the application of statistical methods to analyse the prognosis of a patient in a standard way.

Historically, such detailed analysis of life expectancy of cancer patients in Chile has not been possible. The National Health System in Chile did not record statistical data that would allow them to be carried out until recently. The regional computer platforms for recording cancer data have just been implemented. The registration of data in these computer platforms has made possible studies of obesity as a risk factor for the development of certain types of cancer [6], and the trend of cancer mortality according to the educational level of the adult population [11]. Similarly, the recoded data on cancer patients has made possible the analysis of the epidemiological transition of cancer, the global burden of cancer, and trends in cancer mortality, and its occurrence in Chile [12].

Since statistics are based on large groups of people, we cannot predict what will happen with a particular patient. This is because of personal differences, differences in treatment, and the patients’ response to it. Nevertheless, statistical measures are useful to establish courses of action in prevention and planning of treatment plans to follow.

A key factor in cancer treatment is early detection because many times cases are detected in advanced phases, when organ function is compromised and cancer cells have spread in the body. Therefore, the detection of cancer in early stages is critical in order to design a treatment plan that will greatly increase the chances of it being effective.

The objective of this study is to analyse the occurrence of cancer in the Valparaiso Region thereby further identifying the survival period. This period is the time elapsing from the date of occurrence to the date of death of a patient who suffers from some type of cancer, and it is registered in the cancer module of the Epidemiology Assistance System (SADEPI for its initials in Spanish).

We are also seeking to identify the occurrences and subsequent death for certain types of cancers in a given region and the age groups associated with it.

## Materials and methods

### Data

The data used was extracted from the SADEPI database, specifically from the Cancer Module, which stores information regarding cancer cases in the provinces of Valparaiso and Petorca and from the Causes of Death module.

The criterion for choosing the set of cancers to be used in the study is based on the locations of the cancers with higher mortality [5] in the region of Valparaiso and the highest number of cases in the Cancer Module. Thus six different cancers were selected (Table 1) and these included malignant stomach tumour, malignant colon tumour, malignant gallbladder tumour, others and unspecified, malignant neoplasm of the pancreas, malignant neoplasm of the trachea, bronchi, and lung, malignant breast tumour. All these together caused 49 % of all cancer deaths between 2010 and 2011.

Table 2 shows that the occurrence distribution of four of these six cancers is not statistically different (by using a *χ*^2^ test of identity of distribution) from those observed worldwide (p = 0.05).

The results of the relationship between the date of occurrence of cancer cases and the date of death of the patients using statistical techniques are presented here. The six types of cancers which are studied are among the mostly frequently occurring in the population and for which there are registries of the patient’s cases, including information regarding the date of occurrence and date of death.

For age analysis at the time of the patient’s occurrence and death date, cases where the date of birth was known were considered.

For each cancer case the *survival period* was calculated as the difference in days between the date of occurrence and the date of death of the investigated patients suffering from different types of cancers. Once this data was obtained, it was separated into quartiles to facilitate the analysis.

## Results

### Survival Time (Table 3)

The first quartile groups 25% of the patients who survived fewer days from the detection of their cancer. For this quartile the obtained results show that the malignant gallbladder tumour caused more rapid deaths from its detection in which patients lived 22.5 days from the date of occurrence. It is followed by malignant neoplasm of the pancreas at 57.0 days, the malignant stomach tumour at 64.5 days, malignant neoplasm of the trachea, bronchi, and lung 131.0 days, malignant breast tumour at 149.0 days, and malignant colon tumour at 185 days.

On the other hand, it indicates the third quartile for malignant tumour of the pancreas 75 % of patients did not survive more than 92.5 days of life from the date of occurrence, and it being the group of cancer with lower figures. They were followed by malignant tumours of the stomach with 343.8 days, malignant tumours of the gallbladder and other as well as unspecified parts of the bile tract with 383.5 days, malignant neoplasms of the trachea, bronchus, and lung with 386.0 days, malignant tumours of the colon with 621.0 days, and malignant tumours of the breast with 667.8 days.

**Figure 1.**
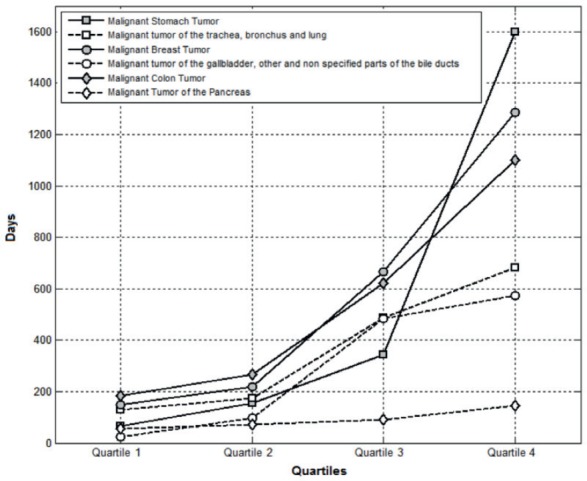
Comparison of life expectancy from date of occurrence to date of death.

Of the reported cases, 100 % of patients (equivalent to quartile four) with malignant pancreatic tumours did not exceed 145.0 days of life since the diagnosis, and it being the cancer that recorded the lowest figures. This is followed by malignant tumours of the gallbladder and others as well as unspecified bile duct at 572.0 days, malignant neoplasms of the trachea, bronchus, and lung at 682.0 days, malignant colon tumours at 1100.0 days , malignant neoplasms of the breast at 1286.0 days, and 1599.0 days for malignant stomach tumours. The lifetimes from the date of occurrence (minimum lifetimes) to death (maximum lifetimes) are shown in Table 3 and a comparison of the lifetime between the dates of occurrence to death divided by quartiles is shown in detail in Figure 1.

### Age at date of occurrence and date of death

**Figure 2.**
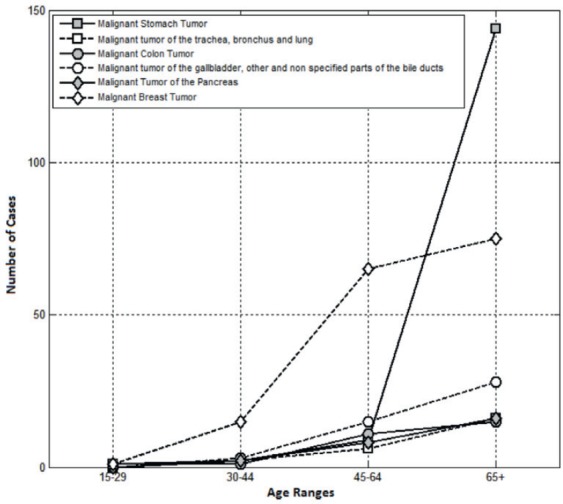
Ages at cancer occurrence.

The age of death was divided into four ranges: 15–29 years of age, 30–44 years of age, 45–64 years of age, and 65 or older. The Figure 2 shows the occurrence of the six types of cancer under study according to the age ranges.

### Stomach (C16) (Table 4)

For malignant tumours of the stomach in the Valparaiso Region in the year 2010, 67.1% of deaths corresponded to men while in the case of women the figures were 32.9%. In the year 2011, there was a decrease in the percentage of cases of men at 66.0% and an increase in women at 34.0%.

For patients between 15–29 years of age there were two cases (0.3%), for patients between 30–44 years of age there were 20 cases (2.6%), for patients between 45–64 years of age 178 cases (23.4%), and for patients 65 and over 560 cases corresponding to 73.7% of the total of cases.

Regarding the age of occurrence, 64.3% of the cases correspond to ages equal to or over 65 years of age, followed by 30.4% in ages 45–64, 3.7% for patients 30–44 years of age, and 1.3% for patients 14–29 years of age.

### Trachea, bronchus, and lung (C33-C34) (Table 5)

In 2010 in the region of Valparaiso 58.6 % of deaths from this cancer corresponded to men, whereas in the case of women the figure was 41.4 %. In 2011 there was an increase in the percentage of cases of men of 63.3% and a decrease in women of 36.7%.

For patients aged 15–29 years no cases were presented, for patients aged 30–44 years 11 cases (1.7 %), 158 cases for patients aged 45–64 (23.9 %), and 492 cases for patients 65 and over corresponding to 74.4% of the total of cases.

Regarding the age of occurrence, 68% of cases are present in ages equal to or over 65, followed by 24% in ages between 46–64 years, and 8.0% for patients ages 30–44.

### Colon (C18) (Table 6)

For malignant colon tumours in the Valparaiso Region in the year 2010, 55.8% of deaths for this type of cancer corresponded to women, while for men the figures where 44.2%. In the year 2011 there was a decrease in the percentage in the case of women with 52.3% and an increase in men with 47.7%

For patients between 15–29 years of age there were three cases (0.8%), for patients aged 30–44 there were five cases (1.3%), for patients aged 45–64 there were 68 cases (18.0%) and for patients aged 65 and over 299 cases corresponding to 79.9% of the total of cases.

Regarding the age of occurrence, 53.5 % of cases occur in or over age 65, followed by a 39.3 % aged 45–64 years, 3.6 % for patients aged 30–44 years, and 14% aged less than 29 years.

### Gallbladder, other parts and non-specified parts of the bile duct (C23-C24) (Table 7)

In the year 2010, malignant tumours of the gallbladder, other parts, and non-specified bile duct registered a total of 173 deaths related to this type of cancer of which 65.9% corresponded to women and 34.1% to men. In 2011 these figures were steady at 66.0% for women and 34.0% for men.

For patients aged 15–29 years one case (0.3 %) occurred for patients aged 30–44 years, 15 cases (4.0 %) for patients aged 45–64, 114 cases (30.3 %) and for patients over 65 years, 246 cases corresponding to 65.4 % of the total of cases.

Regarding the age of occurrence 60.9 % of cases occur in or over age 65, followed by 32.6 % aged 45–64 years, and 6.5 % for patients aged 30–44 years.

### Pancreas (C25) (Table 8)

For malignant pancreatic tumours, in the Valparaiso Region in the year 2010, deaths because of this type of cancer were 43.4% in men and in the case of women this figure was 56.6%. In 2011 there was an increase in the cases pertaining to men with 47.4% and a decrease in women with 52.6%.

For patients aged 15–29 years no cases were presented, for patients aged 30–44 years five cases (1.7 %), for patients aged 45–64 years 90 cases (30.1 %), and patients 65 and over 204 cases corresponding to 68.2 % of the total of cases.

Regarding the age of occurrence 61.5 % of cases occur in or above age 65, followed by a 30.8 % for patients aged 45–64 years, 7.7% for patients 30–44 years, and 0% for patients aged 14–29 years.

### Breast (C50) (Table 9)

For breast cancer in the Valparaiso Region in the year 2010, 99.4% of deaths corresponded to women while in case of men were only 0.6%. In the year 2011 there was an increase in case of men with 1.9% and a decrease in case of women cases to 98.1%.

The age of death was divided into four ranges, patients aged 15–29 years showed one case (0.3 %), patients aged 30–44 years 20 cases (6.2 %), patients aged 45–64 years 119 cases (36.7 %), and patients aged 65 years, over 184 cases corresponding to 56.8 % of the total of cases.

Regarding the age of occurrence 52.6 % of cases occur in or above age 65, followed by a 38.0 % for patients aged 45–64 years, 8.8 % for patients 30–44 years, and 0.6 % for patients aged 14–29 years.

## Discussion

The short survival times from the occurrence of cancer to the death of the patient show that early detection plans, control, and monitoring of risk factors (where relevant) to improve the quality and life expectancy of the patients are necessary.

The results of the study show that one of the cancers with shortest survival time from occurrence is malignant tumour of the gallbladder which is present more frequently in countries of the Andean Zone [12] such as Chile. This cancer is usually detected when a person presents signs or symptoms which indicate that the illness is in its advanced stages. This contrasts with breast cancer which is one of the cancers with highest survival although considered to be one of the most frequent cancers and main causes of death in woman globally [13]. It multiplyiies several times the survival time obtained by malignant gallbladder tumours. Some epidemiological studies have already identified ethnic disparities like the high occurrence in American Indians who have malignant tumours of the gallbladder [14], showing in particular [15] high rates in Chilean Mapuche Indians [16].

Contrary to the present study, these articles show that among Chilean women, gallbladder cancer is the first cause of cancer death, more frequent than breast, lung, and cervical cancers. We notice here that the gallbladder carcinoma is the second cause after the breast cancer and about the same statistical level than lung and stomach cancers. This high prevalence in women (not observed in men) is due probably to a combination of genetic factors, multiparity, or elevated BMI classically associated with a higher risk of developing gallbladder carcinoma [17].

As for the age of death and occurrence of malignant tumours of the stomach, colon as well as trachea, bronchus, and lung, the analysis shows that in Chile, as in other parts of the world [18–20], the majority of affected patients are around 65 years of age. The occurrence decreases in younger adults under 45 years of age [21–23] where cases in general are usually caused by a genetic predisposition.

## Conclusion

The specificities of the cancer situation in Chile are related to the educational policy for cancer prevention, for which specific information, screening, and preventive therapies have been recommended:

- Chile has implemented restrictions on tobacco availability to minors [24].
- Chile has launched screening programmes of early detection of cholelithiasis as this is found in approximately 85% of people with gallbladder cancer [25]. This has led to systematic cholecystectomy or ultrasonic therapy after a nationwide gallbladder cancer study [26–27] done by US NIH, Chilean Regional Hospitals of Antofagasta, Concepcion and Temuco, and the Hospital Sotero del Rio of Santiago. The study also showed that the risk populations in Chile all share a high prevalence of gallstones and/or *Salmonella typhi* infections favouring cholelithiasis.
- Chile has improved the typhoid fever vaccination because it has been thought that typhoidic endemy during the 70s could have affected the occurrence of cancer of the gallbladder [28].
- Chile is edicting public health recommendations rules with specific food labelling indicating the danger of sugar and fat in processed food to prevent obesity, a disease frequently associated with cancer (see for example [29]).

These current and some other future efforts are required to promote prevention and early detection of cancers in order to allow both improvement of health education level, empowerment, and timely access to treatment by affected patients.

## Figures and Tables

**Table 1.** Number of deaths in the Valparaiso Region for six selected types of cancer.

Diagnosis	2010			2011		
	**Both Sexes**	**Men**	**Women**	**Both Sexes**	**Men**	**Women**
Stomach	401	269	132	359	237	122
Trachea, bronchus, lung	307	180	127	354	224	130
Colon	181	80	101	193	92	101
Gallbladder, other and non specified parts of the bile ducts	173	59	114	203	69	134
Pancreas	166	72	94	133	63	70
Breast	166	1	165	158	3	155

**Table 2.** Comparison to world distribution of the most frequent cancers (source: Cancer Research UK) [13].

	In the reference population Mortality 2012		In the sample Mean mortality 2010–11	
**Modalities**	**Population size**	**%**	**Sample size**	**%**
Stomach	723027	31.9%	760	43.3%
Colon	693881	30.6%	374	21.3%
Pancreas	330372	14.6%	299	17.0%
Breast	521817	23.0%	324	18.4%
Total	2269097	100.0%	1757	100.0%
			**Sample representative**	
Modality Nb		4	***χ*^2^-test 3-ddl (p = 0.05)**	

**Table 3.** Distributions with quartiles details of survival times in the Valparaiso Region for six selected types of cancer.

Cancer	Average	Min	Max	Quartile 1	Quartile 2	Quartile 3	Quartile 4
**Malignant Stomach Tumour**	275.2	6.0	1599.0	64,5	156.5	343.8	1599.0
**Malignant tumour of the trachea,** **bronchus and lung**	296.9	21.0	682.0	131.0	175.5	486.0	682.0
**Malignant Breast Tumour**	263.1	6.0	1286.0	149.8	218.0	667.8	1286.0
**Malignant tumour of the gallbladder, other and non specified parts of the bile ducts**	240.7	4.0	572.0	22.5	97.0	483.5	572.0
**Malignant Colon Tumour**	398.0	4.0	1100.0	185.0	266.0	621.0	1100.0
**Malignant tumour of the Pancreas.**	77.5	20.0	145.0	57.0	72.5	92.5	145.0

**Table 4.** Number of deaths caused by malignant stomach tumour (C16).

Age	15–29	30–44	45–64	65 and more
Feminine	2	10	50	192
Masculine	0	10	128	368
Totals	2	20	178	560

**Table 5.** Number of deaths caused by malignant tumour of the trachea, bronchus, and lung (C33-C34)

Age	15–29	30–44	45–64	65 and more
Feminine	0	6	54	197
Masculine	0	5	104	295
Totals	0	11	158	492

**Table 6.** Number of deaths caused by malignant colon tumour (C18)

Age	15–29	30–44	45–64	65 and more
Feminine	1	4	33	164
Masculine	2	1	34	135
Totals	3	5	67	299

**Table 7.** Number of deaths caused by malignant tumour of the gallbladder, other and non specified parts of the bile ducts (C23-C24)

Age	15–29	30–44	45–64	65 and more
Feminine	0	10	73	165
Masculine	1	5	41	81
Totals	1	15	114	246

**Table 8.** Number of deaths caused by malignant tumour of the pancreas (C25).

Age	15–29	30–44	45–64	65 and more
Feminine	0	3	45	116
Masculine	0	2	45	88
Totals	0	5	90	204

**Table 9.** Number of deaths caused by malignant breast tumour (C50).

Age	15–29	30–44	45–64	65 and more
Feminine	1	19	118	182
Masculine	0	1	1	2
Totals	1	20	119	184

## References

[1] Rothwell PM (2012). Short-term effects of daily aspirin on cancer incidence, mortality, and non-vascular death: analysis of the time course of risks and benefits in 51 randomised controlled trials. Lancet.

[2] Ferlay J (2007). Estimates of the cancer incidence and mortality in Europe in 2006. Ann Oncol.

[3] Jemal A (2010). Cancer Statistics, 2010. CA Cancer J Clin.

[4] Principales Causas de Muerte en Chile por Regiones 1997–2003. http://www.ine.cl/canales/chile_estadistico/demografia_y_vitales/estadisticas_vitales/pdf/causas_de_muerte_regiones%202003.PDF.

[5] Medina E, Kaempffer AM (2001). Mortalidad por cáncer en Chile: consideraciones epidemiológicas. Rev Med Chil.

[6] Luisa Garmendia M, Ruiz P, Uauy R (2013). Obesity and cancer in Chile: Estimation of population attributable fractions. Rev Med Chil.

[7] Roco Á (2013). Cancer Status in Chile 2000-2010. Medico Sociales.

[8] Ferlay J (2013). Cancer incidence and mortality patterns in Europe: estimates for 40 countries in 2012. Euro J Cancer.

[9] Ferlay J (2010). Estimates of worldwide burden of cancer in 2008: GLOBOCAN 2008. Inter J Cancer.

[10] National Cancer Institute, Understanding Cancer Prognosis (2014). http://www.cancer.gov/about-cancer/diagnosis-staging/prognosis.

[11] Herrera Riquelme CA (2015). Tendencia de la mortalidad por cáncer en Chile según diferencias por nivel educacional, 2000-2010. Rev Panam Salud Publica.

[12] Itriago GL, Silva IN, Cortes FG (2013). Epidemiology of cancer in Chile and Worldwide: present and future. Revista Médica Clínica Las Condes.

[13] Cancer Research UK, Cancer incidence for common cancers (2013). http://www.cancerresearchuk.org/health-professional/cancer-statistics/incidence/common-cancers-compared#heading-Zero.

[14] Hundal R, Shaffer EA (2014). Gallbladder cancer: epidemiology and outcome. Clin Epidemiol.

[15] Shaffer EA (2008). Gallbladder cancer: the basics. Gastroenterol Hepatol.

[16] Lazcano-Ponce EC (2001). Epidemiology and molecular pathology of gallbladder cancer. CA: A Cancer J Clin.

[17] Serra I (1988). Perspectivas del cáncer biliar y otros cánceres importantes en Chile. Cuad Méd Soc (Santiago de Chile).

[18] Ministerio de SaludGobierno de Chile, GuíaClínicaCáncerGástrico (2010). http://web.minsal.cl/portal/url/item/722233c6b943cd08e04001011f011d5e.pdf.

[19] Santos-Martínez MJ (2005). Características del cáncer de pulmón en un hospital universitario. Cambios epidemiológicos e histológicos en relación con una serie histórica. Arch Bronconeumol.

[20] Potter JD (1993). Colon cancer: a review of the epidemiology. Epidemiologic Rev.

[21] Pisano R (1990). Cáncer Gástrico en la provincia de Valdivia (Chile) 1977-1987. Rev Méd Chil.

[22] Eguchi J, Fujii M (1999). Gastric cancer in young patients. J Am Coll Surg.

[23] Ezzati M (2005). Role of smoking in global and regional cancer epidemiology: current patterns and data needs. Int J Cancer.

[24] de la Jara JJ (2015). A snapshot of cancer in Chile: analytical frameworks for developing a cancer policy. Biol Res.

[25] Randi G, Franceschi S, La Vecchia C (2006). Gallbladder cancer worldwide: geographical distribution and risk factors. Inter J Cancer.

[26] Wistuba II, Gazdar AF (2004). Gallbladder cancer: lessons from a rare tumour. Nat Rev Cancer.

[27] Miquel JF (1998). Genetic epidemiology of cholesterol cholelithiasis among Chilean Hispanics, Amerindians, and Maoris. Gastroenterology.

[28] Ferreccio C, Khan AA (2011). Salmonella typhi and gallbladder cancer in:. Bacteria and Cancer.

[29] Chile seeks to fight obesity with new food labeling law. http://bigstory.ap.org/article/f9b43cf296a546a09ef1c11d5e3fec01/chile-seeks-fight-obesity-new-food-labeling-law.

